# The Intersectionality of Person, Space, and Time for Understanding Aging in Place

**DOI:** 10.1093/geroni/igaa057.1409

**Published:** 2020-12-16

**Authors:** Widya Ramadhani, Maurita Harris, Wendy Rogers

**Affiliations:** University of Illinois at Urbana-Champaign, Urbana, Illinois, United States

## Abstract

Aging in place is interpreted differently across times and disciplines in the literature. Multiple interpretations of aging in place can lead to differences in expectations and goals when planning products, services, and technologies for older adults. We conducted a historical review across databases in the fields of anthropology, architecture, gerontol-ogy, medicine, psychology, and sociology to explore the evolution of ‘aging in place’ term across time and disciplines. We included articles that used the terminology “aging in place” or “ageing in place” in titles, abstracts, keywords, or subject. From the aging in place definition excerpts collected, we identified the preliminary themes and grouped them into three main themes: people, space, and time. Although the narrative of aging in place is highly related to living spaces, the cause and influencing factors are tied beyond the space. Person and time-related factors that are related to the aging experience im-pact the way aging in place is defined. When designing products, services, and technolo-gies to support successful aging in place, designers, researchers, policymakers, and care-givers should be aware that aging in place is at the intersection of personal, spatial, and temporal elements of older adults’ lives. Based on the multiple perspectives of disci-plines, we concluded that aging in place is beyond the matter of location, but also takes into account the person’s capacity and the changes over the person’s lifespan. Founda-tional understanding of the multiple factors that influence aging in place is critical to support older adults to have a healthy and optimal aging experience.

